# As recomendações em saúde pública como microagressões: varíola dos macacos e populações LGBTQIA+ 

**DOI:** 10.1590/0102-311XPT020623

**Published:** 2023-11-10

**Authors:** Douglas Antonio Rocha Pinheiro, Alexandre Gustavo Melo Franco de Moraes Bahia

**Affiliations:** 1 Faculdade de Direito, Universidade de Brasília, Brasília, Brasil.; 2 Departamento de Direito, Universidade Federal de Ouro Preto, Ouro Preto, Brasil.

**Keywords:** Estigma Social, Direitos Sexuais, Pessoas LGBTQIA+, Varíola dos Macacos, Social Stigma, Reproductive Rights, LGBTQ Persons, Monkeypox, Estigma Social, Derechos Sexuales, Personas LGBTQ, Viruela del Mono

## Abstract

O artigo tem por objetivo analisar recomendações de saúde pública da Organização Mundial da Saúde e das manifestações de seu Diretor-geral durante a epidemia de varíola dos macacos endereçadas a homens que fazem sexo com homens (HSH) à luz da microagressão como categoria de análise. Questiona-se o potencial estigmatizador de divulgação estatística, para público amplo, de que 98% dos infectados estavam entre HSH, bem como a utilização da própria categoria HSH e da sugestão de abstinência sexual parcial ou total como forma de interromper a disseminação viral. Sugere-se como alternativas capazes de, simultaneamente, garantir políticas de prevenção de doenças sem estigmatizar grupos vulneráveis, especialmente a população LGBTQIA+: (i) diferenciar as divulgações voltadas ao público geral das destinadas às populações predominantemente contaminadas e sujeitas a maior grau de vulnerabilidade social; (ii) superar a utilização da expressão HSH para, nas comunicações destinadas à ampla audiência, utilizar a expressão SGD (população sexo e gênero diversa), mantendo-se o procedimento de registrar, nas pesquisas científicas e nos formulários de atendimento, a identidade de gênero e a orientação sexual por autodeclaração dos pacientes; (iii) evitar mensagens que abordem a sexualidade de modo negativo, reforcem uma vivência sexual majoritária e gerem uma responsabilização socialmente punitiva do infectado, excluindo, pois, das recomendações voltadas ao público amplo a sugestão de abstinência sexual parcial, relativa à redução do número de parceiros, ou de abstinência sexual total, exceto para os casos de pessoas na fase ativa da infecção ou no período imediato à recuperação.

## Abstract (en)

The article aims to analyze public health recommendations of the World Health Organization and the manifestations of its Director-General during the mpox epidemic addressed to men who have sex with men (MSM) in the light of microaggression as a category of analysis. The stigmatizing potential of statistical disclosure to a broad public that 98% of those infected were among MSM is questioned, as well as the use of the MSM category itself and the suggestion of partial or total sexual abstinence as a way to stop viral dissemination. The following are suggested as alternatives capable of simultaneously guaranteeing disease prevention policies without stigmatizing vulnerable groups, especially the LGBTQIA+ population: (i) differentiating the disclosures addressed to the general public from those aimed at populations predominantly contaminated and subject to a higher degree of social vulnerability; (ii) overcoming the use of the expression MSM, in communications aimed at a wide audience, to use the expression SGD (sex and gender diverse population), maintaining the procedure of recording, in scientific research and in medical forms, the gender identity and sexual orientation by self-declaration of patients; (iii) avoiding messages that negatively address sexuality, reinforce a majority sexual experience, and generate a socially punitive responsibility of the infected, thus excluding from the recommendations aimed at the broad public, the suggestion of partial sexual abstinence, related to the reduction of the number of partners, or total sexual abstinence, except for the cases of people in the active phase of infection or in the immediate period of recovery.

## Resumen

Este artículo tiene como objetivo analizar las recomendaciones de salud pública de la Organización Mundial de la Salud y las manifestaciones de su Director General durante la epidemia de viruela del mono, dirigidas a los hombres que tienen relaciones sexuales con hombres (HSH), a la luz de la microagresión como categoría de análisis. Se discute el potencial estigma de la divulgación estadística para una amplia audiencia por haber informado que el 98% de los infectados estaban entre HSH, así como el uso de la categoría HSH en sí y la sugerencia de abstinencia sexual parcial o total como forma de detener la propagación viral. Se sugieren como alternativas capaces de garantizar simultáneamente políticas de prevención de enfermedades sin estigmatizar a los grupos vulnerables, especialmente la población LGBTQIA+: (i) distinguir las divulgaciones dirigidas al público en general de las destinadas a poblaciones predominantemente contaminadas y sujetas a un mayor grado de vulnerabilidad social; (ii) dejar de usar el término HSH para, en comunicaciones dirigidas a la amplia audiencia, emplear el SGD (población sexo y género diversa), manteniendo el procedimiento de registro (en investigaciones científicas y formularios de atención) de la identidad de género y la orientación sexual por autodeclaración de los pacientes; (iii) evitar mensajes que tratan la sexualidad de manera negativa, que aseguran una experiencia sexual mayoritaria y que generan una rendición de cuentas socialmente punitiva de los infectados, excluyendo, por lo tanto, de las recomendaciones dirigidas al público en general la sugerencia de abstinencia sexual parcial, relativa a la reducción del número de parejas, o de abstinencia total, excepto en los casos de estadio activo de la infección o del período inmediato de recuperación.

## Introdução

Em 23 de julho de 2022, a Organização Mundial da Saúde (OMS) declarou a varíola dos macacos como Emergência de Saúde Pública de Importância Internacional (ESPII), por se tratar de um evento inesperado e significativo capaz de acarretar implicações à saúde pública de diversos países por meio da propagação transnacional de doenças, exigindo, potencialmente, uma resposta internacional coordenada ^1^. Quatro dias depois, Tedros Adhanom Ghebreyesus, Diretor-geral da OMS, decidiu explicar o modo como o surto de varíola dos macacos poderia ser contido. Por meio de uma coletiva de imprensa virtual, transmitida de Genebra (Suíça), ele alertou que o modo mais eficaz de interromper a disseminação da doença seria reduzir o risco de exposição, ou seja, levar as pessoas a tomarem decisões seguras para si e para os demais. Após a indicação de tal solução genérica, Ghebreyesus fez uma recomendação particular a um grupo específico de pessoas: “*para homens que fazem sexo com homens, isso inclui* (...) *reduzir o número de parceiros sexuais, reconsiderar o sexo com novos parceiros e trocar informações de contato com quaisquer novos parceiros para possibilitar, se necessário, o monitoramento*” ^2^. Por fim, acrescentou que, embora homens que fazem sexo com homens (HSH) correspondessem a 98% do total de casos registrados, qualquer pessoa exposta ao vírus causador da varíola dos macacos (vírus Monkeypox - MPXV) poderia se contaminar, razão por que era necessário resguardar os grupos de pessoas mais vulnerabilizadas, tais como crianças, gestantes e imunodeprimidos ^2^.

Em 25 de maio de 2022, a própria OMS já havia emitido uma recomendação sobre saúde pública para gays, bissexuais e HSH, na qual indicava que o modo de proteger a si e aos outros incluía: (i) isolar-se em casa e conversar com um profissional da saúde se apresentasse sintomas; (ii) limpar mãos, objetos e superfícies que fossem tocados com frequência; (iii) usar máscara, caso mantivesse contato com alguém que apresentasse sintomas; e (iv) evitar contato de pele ou face, inclusive contato sexual, com qualquer pessoa que apresentasse sintomas ^3^. Nota-se a ausência de qualquer menção à redução de parceiros sexuais. Porém, em uma atualização de tal recomendação ^4^, emitida em 18 de julho de 2022, passaram a constar outras sugestões de condutas: evitar contato sexual se houvesse erupções cutâneas novas e incomuns, ao menos até ser testado para MPXV e para infecções sexualmente transmissíveis (ISTs); trocar dados de contato com pessoas com quem tenha mantido relação sexual, mesmo que casual, e reduzir o número de parceiros sexuais. Embora essa nova recomendação não fizesse qualquer menção ao percentual de casos entre pessoas gays, bissexuais ou HSH, a convergência entre tal documento e as orientações dadas oralmente pelo Diretor-geral da OMS na mencionada coletiva de imprensa possivelmente indica que a estatística foi levada em conta na sua elaboração.

A ênfase na orientação endereçada quase exclusivamente a HSH, com destaque para a divulgação do alto percentual estatístico de infectados nesse grupo, apesar da informação com ressalvas de que o estigma e a discriminação seriam tão perigosos quanto o vírus na disseminação da doença ^2^^,^^3^^,^^4^, despertou um receio considerável na comunidade científica. Surgiram vários alertas de que a conduta questionável da OMS em tal cenário de ESPII pudesse reavivar a patologização de práticas, orientações e identidades dissidentes de sexualidade e gênero, reforçar posturas homofóbicas/racistas e repetir os discursos persecutórios que atingiam pessoas vivendo com HIV, especialmente nas décadas de 1980 e 1990 ^5^^,^^6^^,^^7^^,^^8^. O receio não é imotivado. Desde que Chester Peirce ^9^ cunhou o termo “microagressão” em contextos de discriminações étnico-raciais, o conceito tem sido utilizado para desvelar insultos verbais, comportamentais e ambientais que, manifestando-se de modo sutil e cotidiano, intencionais ou inconscientes, comunicam à pessoa ou ao grupo-alvo uma mensagem hostil, depreciativa e negativa ^10^. As microagressões costumam se manifestar, em termos gerais, de três modos: por microassalto, nas discriminações explícitas que pretendem inferiorizar o outro por meio de rótulos, comportamentos ou símbolos, fazendo-o se sentir constrangido, a fim de criar dificuldades adicionais à sua permanência nos espaços sociais; por microinsultos, nos discursos sutis que, mesmo reproduzidos de modo irrefletido, mostram falta de sensibilidade, de acolhimento e de cuidado com o passado histórico ou com a identidade das pessoas ofendidas; por microinvalidação, nas narrativas que excluem, negam ou invalidam pensamentos, sentimentos e experiências das pessoas agredidas ^10^.

Para além dessa tríplice manifestação, têm sido levantadas formas específicas de microagressão sofridas pelas coletividades mais vulnerabilizadas, tais como pessoas negras, indígenas, mulheres, pessoas com deficiência e populações LGBTQIA+. Quanto ao último grupo, que interessa a este artigo, identificaram-se alguns temas que servem como aglutinadores para a compreensão das microagressões relativas à orientação sexual e à identidade de gênero ^11^^,^^12^: (i) uso de terminologia transfóbica ou heterossexista, quando, por exemplo, não são respeitados o nome social ou os pronomes de gênero com que as pessoas se identificam; (ii) respaldo de determinado padrão comportamental ou cultural cis-heteronormativo, criando-se expectativas uniformizantes em relação a certo modo de agir, de viver e de se relacionar e, ao mesmo tempo, reprovando moralmente quem não corresponda a tal padrão; (iii) presunção de uniformidade entre pessoas dissidentes de sexualidade e de gênero, o que anula as experiências individuais em detrimento de um estereótipo socialmente cristalizado; (iv) exoticização, quando as minorias referidas são desumanizadas ou tratadas como objeto de curiosidade; (v) desaprovação ou desconforto com a experiência não normativa, quando a interação com pessoas e práticas sexo e gênero diversas causa nos demais um incômodo em grau suficiente para motivar reações pessoais, coletivas e institucionais invisibilizantes; (vi) negação da homotransfobia e do heterossexismo sociais, quando, por exemplo, comportamentos hegemônicos excludentes são considerados neutros e isonômicos, atribuindo-se à vítima, de modo indevido, certo transtorno delirante de tipo persecutório; (vii) patologização da não normatividade, deslocando a diversidade social para o campo médico, psiquiátrico ou farmacológico; (viii) negação da homotransfobia e do heterossexismo individuais, diante da recusa das pessoas em autorreconhecer seu preconceito ou sua percepção normatizadora, mesmo quando alertadas pela vítima da microagressão; (ix) subcidadanização ou cidadania de segunda classe, práticas, políticas e normas que reforçam privilégios de pessoas cis-hetero em detrimento daquelas não normativas; (x) hipersexualização, quando pessoas dissidentes de sexualidade e de gênero são consideradas promíscuas, segundo padrões morais hegemônicos, ou reduzidas em sua complexidade e consideradas apenas em relação ao seu comportamento sexual; e (xi) pânico homotransfóbico, quando se evitam interações com pessoas sexo e gênero diversas com base no medo internalizado de que isso possa modificar a própria identidade de gênero/orientação sexual ou de terceiros, como crianças e adolescentes.

A microagressão não é uma experiência menor de discriminação. No decorrer dos anos, reações conservadoras tentaram vinculá-la ao suposto patrulhamento social motivado pela linguagem politicamente correta, justificá-la em nome da liberdade de manifestação de pensamento, desacreditá-la ao apontarem a dificuldade de mensuração dos danos psíquicos por ela causados ou deslegitimá-la por supostamente fomentar uma cultura da vitimização, cuja consequência seria a fragilização dos jovens diante dos desafios da vida adulta. Por tais motivos, já se cogitou substituir a terminologia microagressões por atos sutis de exclusão, a fim de se tirar o foco do radical “micro” como algo insignificante e deixar ainda mais claro que tal linguagem excludente é performativa. Afinal, em vez de se restringir ao campo das palavras, ela não só fomenta como se converte em atos concretos de subcidadanização ou, no limite, de exclusão de pessoas vulnerabilizadas e estigmatizadas ^13^. Microagressões não são fatos pontuais e isolados, mas sim ações e discursos recorrentes e acumulativos, que criam condições para estigmatizações que, no campo da Saúde Coletiva, podem afetar a pessoa necessitada de assistência à saúde, o profissional que a recepciona nos serviços de atenção primária ou especializada à saúde, os próprios órgãos encarregados de elaboração de protocolos e recomendações em saúde pública. Por esse motivo, é importante tê-las em mente para que as formas de comunicação sejam cada vez mais inclusivas, o que no campo da Saúde Coletiva significa, concretamente, salvar mais vidas.

A hipótese deste ensaio, considerando a reação imediata da comunidade científica, preocupada com a atuação potencialmente estigmatizadora da OMS no caso “*monkeypox*”, especialmente em relação a HSH, é de que nesse caso as recomendações em saúde pública destinadas à população LGBTQIA+ apresentavam elementos de natureza microagressiva. Com isso, podem ter sido criados contextos inadequados de constrangimento, com potenciais consequências psicossociais à pessoa cuja saúde se buscava inicialmente proteger. Os objetivos que se pretende alcançar com este texto são apontar como tais microagressões correspondem a expressões consolidadas nos programas de saúde pública e propor alternativas capazes de, simultaneamente, garantir políticas de prevenção de doenças sem estigmatizar grupos vulnerabilizados. Para tanto, vale-se da perspectiva das Ciências Sociais em Saúde, a fim de desvelar processos hegemônicos heterocisnormativos que dificultam às populações LGBTQIA+ o exercício do direito à sexualidade e à saúde sexual.

## A estatística microagressiva

Em 1958, a varíola dos macacos foi identificada pela primeira vez: macacos pertencentes ao Instituto Statens Serum, um centro de pesquisas sobre doenças infecciosas localizado em Copenhague (Dinamarca), foram diagnosticados com uma doença viral inédita semelhante à varíola comum ^14^. A zoonose, assim, acabou vinculada aos primatas, embora roedores fossem seus vetores mais usuais. Os primeiros casos de infecção humana só foram reportados nos anos de 1970 e 1971 nas áreas rurais de Serra Leoa, Libéria, Nigéria e República Democrática do Congo ^15^^,^^16^. Com o tempo, a varíola dos macacos se tornou endêmica na parte central e ocidental do continente africano, apresentando alguns picos de contaminação, como o surto ocorrido em 1996 e 1997 na República Democrática do Congo ^17^. Na Nigéria, que desde 1978 não registrava casos de contágio humano, a varíola dos macacos reemergiu em 2017 ^18^. Em maio de 2022, o Reino Unido identificou a doença em um viajante proveniente da Nigéria ^19^. No início do mês seguinte, 27 países de regiões não endêmicas já confirmavam casos de varíola dos macacos ^20^. O perfil desse surto, marcado pela transmissão do vírus entre humanos sem a necessidade de um vetor, aliado à intenção de evitar o estigma na linguagem, fez inclusive com que a OMS sugerisse, em 28 de novembro de 2022, a substituição do termo “*monkeypox*” por mpox, resguardando um período de transição de um ano em que ambos os significantes serão admitidos ^21^. Essa sugestão, porém, ainda não foi ratificada pelo Comitê Internacional de Taxonomia de Vírus (ICTV), organização encarregada de uniformizar a classificação universal dos vírus.

Diante do surto crescente, em junho de 2022, um grupo de pesquisadores vinculados ao SHARE (Sexual Health and HIV All East Research), sediado em Londres (Reino Unido), a que se juntaram pares de outros continentes com o objetivo de se ampliar o mapeamento global dos casos reportados, analisaram um total de 528 casos comprovados de infecção humana, distribuídos em 16 países. O estudo ^22^, publicado originariamente em 21 de julho de 2022, consolidou o dado estatístico que dias depois seria mencionado pelo Diretor-geral da OMS: 98% dos contaminados eram homens gays e bissexuais. Obviamente, a conclusão não se restringia a isso. Uma boa parte do artigo se concentrou no diagnóstico e nos aspectos clínicos da mpox, enfatizando que em 95% dos casos observavam-se erupções ou lesões de pele, majoritariamente encontradas na região anogenital, além de outros sintomas percentualmente menos recorrentes: febre (62%), linfadenopatia (56%), letargia (41%), mialgia (31%) e dor de cabeça (27%). Porém, grande parte dos dados levantados pelo estudo referiam-se aos aspectos clínicos e demográficos das pessoas contaminadas, tais como: idade, orientação sexual, sexo ou gênero, raça ou etnia, viver com HIV, número de parceiros sexuais no último trimestre, recorrência da prática sexual sob influência de drogas psicoativas (*chemsex*). O marcador étnico-racial é oportuno para demonstrar um dos vieses do estudo. Afinal, o dado de que 75% dos infectados eram autodeclarados brancos e só 5% autodeclarados negros não pode ser separado do recorte geográfico da origem dos casos analisados. Os pesquisadores foram transparentes ao indicar a origem majoritariamente europeia dos casos, a delimitação estrita entre os países das Américas (Canadá, Estados Unidos, México e Argentina, apenas) e a inexistência de dados relativos a países africanos. Ainda assim, é surpreendente que as imagens das lesões cutâneas presentes no estudo apresentem de modo paritário homens brancos e negros. Embora isso possa ser estratégico para se evitar uma vinculação imagética imediata entre a patologia e o marcador étnico-racial, tal escolha parece dissonante em relação à intencionalidade do recorte proposto.

Um outro viés que não pode ser desconsiderado e que pode ter interferido no alto percentual relatado entre HSH refere-se às clínicas nas quais a infecção foi confirmada por meio de plataformas PCR (reação em cadeia da polimerase), critério utilizado pelos pesquisadores para confiabilidade de diagnósticos dos casos estudados. Mais da metade deles tiveram origem em clínicas especializadas em tratamentos de HIV/aids (29%) e de saúde sexual (23%). Combinados com o perfil dos pacientes - 41% de pessoas vivendo com HIV e 33% de usuários de profilaxia pré-exposição ao HIV (PrEP) -, praticamente três quartos dos casos reportados são de pacientes que mantêm uma prática de acompanhamento médico-hospitalar regular, portanto, mais sujeitos a uma identificação rápida da mpox ^22^. Tal aspecto precisa ser devidamente enfatizado por dois motivos: primeiro, pela confiabilidade necessária que um suposto infectado precisa ter no seu clínico para se submeter a um diagnóstico de infecção socialmente estigmatizada; segundo, pela grande quantidade de ISTs que podem imitar a varíola dos macacos, tais como herpes, clamídia, gonorreia e sífilis, especialmente nos casos em que as erupções ou lesões cutâneas tenham surgido em menor número e em regiões específicas do corpo ^23^. Tal viés não passou despercebido pelos pesquisadores do SHARE, que o indicaram como uma ressalva do próprio estudo em um breve parágrafo ao fim do artigo, mas não o fizeram constar no próprio resumo da pesquisa. Com isso, especialmente por meio da câmara de eco fornecida pela própria OMS por meio do seu Diretor-geral, disseminou-se a questionável informação do altíssimo percentual de contaminados, os tais 98%, entre HSH.

De duas, uma: se o viés apresentado pode ter majorado sobremaneira o percentual de ocorrência da varíola dos macacos entre HSH em relação ao total de casos reportados, seja em razão de os locais que disponibilizaram os dados realizarem acompanhamento continuado de várias pessoas com esse perfil ou devido à subnotificação entre pessoas heterossexuais não habituadas a procurar o sistema de saúde com a mesma regularidade, a divulgação de tal estatística pela OMS deveria ter sido evitada para não minorar a importância da profilaxia entre a população geral. No Brasil, por exemplo, mesmo considerando os mais de 30 anos da pandemia de HIV/aids, bem como os dados oficiais recentes de que homens heterossexuais respondem por 49% dos casos, enquanto os homossexuais representam 38% deles, o diagnóstico para os primeiros ainda é recebido como algo inesperado e surpreendente. Essa surpresa decorre do imaginário social que relacionou determinadas infecções a, sucessivamente, grupos de risco, comportamentos de risco e populações-chave ^24^. Porém, se o viés não interferiu no resultado e o percentual de pessoas contaminadas pela mpox for mesmo composto quase integralmente por HSH, a recomendação em saúde pública proferida pelo Diretor-geral da OMS na coletiva de imprensa e endereçada à população geral é uma microagressão, tanto por respaldar determinado padrão comportamental ou cultural cis-heteronormativo, vinculando-o à saúde, quanto por patologizar a não normatividade sexual. Longe de se tornar eficaz na profilaxia da infecção, esse comunicado acaba estigmatizando as pessoas que precisam de atenção à saúde.

Desse modo, como conclusão parcial, os órgãos internacionais e estatais responsáveis pelas recomendações em saúde pública relativas a ISTs que sejam majoritariamente reportadas pelas populações LGBTQIA+ deveriam se pautar por duas condutas: quanto ao público geral, manter apenas as divulgações relacionadas aos sintomas, às formas de contágio e de profilaxia; e com relação às populações predominantemente contaminadas e sujeitas a maior grau de vulnerabilidade social, realizar divulgações específicas e focais. Tais divulgações diferenciadas seriam feitas diretamente nos locais usualmente frequentados para a prática de sexo casual e nos serviços ambulatoriais de atendimento a ISTs, virtualmente nos aplicativos de relacionamento mais utilizados, a exemplo da parceria acordada entre o Grindr e a Agência de Segurança Sanitária do Reino Unido (UKHSA) para envio de alertas sobre a varíola dos macacos aos seus usuários ^8^. De modo adicional, as divulgações poderiam ser realizadas por meio de associações e organizações não governamentais que mantenham contato mais amigável e menos disciplinar que os órgãos estatais com essas populações. Um exemplo da urgência de ações específicas para coletivos potencialmente mais vulnerabilizados é a experiência dos Estados Unidos com os surtos regulares de doença meningocócica invasiva entre HSH, especialmente atribuídos ao sorogrupo C. Há algum tempo, identificou-se não só a necessidade de se pensar em modos efetivos de ampliar a cobertura vacinal em tal grupo, mas de lastrear as especificidades relacionadas a HSH jovens e negros, proporcionalmente mais atingidos pela meningite e não efetivamente alcançados pelas estratégias gerais traçadas pela vigilância sanitária ^25^.

## A categoria microagressiva

A questionável divulgação ampla do percentual elevado de contaminação por MPXV entre pessoas sujeitas à estigmatização social não foi a única microagressão feita por Ghebreyesus. A utilização da categoria HSH pelo Diretor-geral da OMS na já mencionada coletiva virtual de imprensa ^2^ não só contradiz o próprio padrão adotado pela organização nas suas recomendações de saúde pública relativas à mpox, sempre referindo-se a gays, bissexuais e HSH ^3^^,^^4^, como também desconsidera a crítica crescente que ela tem sofrido por pesquisadores em saúde coletiva ^26^^,^^27^^,^^28^^,^^29^. É verdade que a menção à categoria HSH não reflete apenas um uso sedimentado que, como expressão, já era mencionada em 1990 ^30^ e, como acrônimo, a partir de 1994 ^31^. Há argumentos que, no decorrer dos anos, justificaram seu uso: (i) argumentos de natureza epidemiológica, considerando que o estudo dos determinantes relacionados às doenças tem relação com os comportamentos do indivíduo que possam colocá-lo em risco de contaminação e não propriamente com sua identidade, o que, nos primeiros anos de surto de HIV/aids, parecia adequado para evitar processos sociais de estigmatização ^26^; (ii) argumentos de fundo geracional, pois, na década de 1980, o termo gay surgiu como uma alternativa política e metodologicamente livre do peso da tradição patologizante da psiquiatria, sendo incorporado por jovens, enquanto o termo homossexual permanecia sendo usado pelos homens mais velhos, o que demandava uma terceira expressão que superasse a divergência etária ^32^; (iii) argumento de caráter englobante, já que o termo gay, nos Estados Unidos, acabou demarcando determinada sexualidade dissidente, mas identitariamente branca, urbana e de classe média, ao passo que HSH, comumente relacionado aos marcadores étnico-raciais e de classe, ampliava o espectro abordado para pretensamente incluir negros, latinos e pobres ^26^.

Com o passar dos anos, porém, dois movimentos aconteceram: um, no âmbito das Ciências da Saúde; outro, no campo social. Nas Ciências da Saúde, a emergência do conceito de vulnerabilidade, originariamente surgido no campo do direito internacional dos direitos humanos ^33^, apontou que as atividades de intervenção em saúde não poderiam se basear prioritariamente na perspectiva individualizante e probabilística do consolidado conceito de risco ^34^. Com isso, a chance de pessoas adoecerem passou a ser pensada por um triplo eixo: o componente individual, relativo à capacidade de cada pessoa internalizar práticas protetivas; o componente social, referente ao contexto circundante de cada indivíduo, tais com acesso a informação, recursos materiais e escolarização; e o componente programático, relativo ao alcance e à efetividade dos programas de saúde pública ^33^. No campo social, o grupo das pessoas sexo e gênero diversas passou por um processo de complexificação, de tal modo que a tímida tentativa de incorporar o termo mulheres que fazem sexo com mulheres aos estudos não foi suficiente. Afinal, há questões de fundo mais desafiadoras. Uma primeira crítica ao uso do termo HSH ^26^ refere-se à desvalorização das conquistas históricas do direito à autoidentificação, cuja importância não decorre apenas dos processos comunitários de empoderamento, autoestima e articulação política para a reivindicação de interesses comuns. Afinal, a superação de uma visão reducionista e estereotipada das expressões de gênero favorece uma atenção integral à saúde que seja sensível à pluralidade das populações LGBTQIA+ no campo das políticas públicas. A título de exemplo, o Centro de Controle e Prevenção de Doenças dos Estados Unidos (CDC), em suas diretrizes para tratamento de ISTs, aponta que o conceito de HSH não é claro sobre a inclusão ou exclusão de pessoas trans, admitindo ambas as possibilidades caso o termo “homem” leve em conta “*o sexo ao nascimento (isto é, com mulheres trans incluídas) ou a atual identidade de gênero (isto é, com homens trans incluídos)*” ^35^ (p. 15). Por isso, o órgão recomenda que sejam sempre incluídos nos prontuários dos pacientes os dados relativos à orientação sexual e à identidade de gênero deles e de seus parceiros, o que põe em dúvida a capacidade de o conceito HSH e os inúmeros estudos que o utilizam como categoria de análise serem suficientes para mapear soluções relativas às políticas de saúde. Ao se considerar que, em níveis globais, pessoas trans enfrentam maiores barreiras de acesso a serviços de atenção à saúde sexual, se comparadas a pessoas cisgênero ^36^, a redução de ambas a uma só categoria abrangente fragiliza a efetividade das soluções profiláticas e de tratamento propostas abstratamente.

A essa primeira crítica endereçada à categoria HSH, somam-se outras: (i) o foco restrito ao comportamento pode gerar tanto uma hipersexualização ^27^, por desconsiderar a integralidade de processos de subjetivação que sofrem influxos de fatores estruturais e contextuais, quanto uma subsexualização, por empobrecer o sentido social da sexualidade ao desconsiderar as variáveis das identidades e dos desejos ^26^. Além disso, pode reforçar (ii) certa exotização, especialmente quando os formulários de pesquisa rastreiam práticas, encontros e formas de prazer sexual, tais como *cruising*, celebrações do orgulho (*pride events*) e *chemsex*, que se distanciam do padrão heteronormativo, e (iii) uma presunção de uniformidade entre pessoas dissidentes de sexualidade e de gênero, generalizando condutas que, em uma subcultura própria, podem dizer respeito apenas a performances determinadas e provisórias. A utilização de uma categoria desvinculada das identidades sociais gera, ainda, (iv) uma dificuldade de endereçamento dos alertas preventivos veiculados pelos órgãos de saúde, na medida em que as pessoas destinatárias de tais mensagens dificilmente com elas se identificam. Um exemplo disso é que, nos Estados Unidos, embora pesquisadores pretendessem que HSH servisse para incluir homens gay negros nas pesquisas epidemiológicas, essa categoria não gera reconhecimento suficiente entre tais destinatários. Homens gays negros ou se aproximam das identidades clássicas vinculadas aos homens brancos (gay e homossexual) ou se autoidentificam por meio de categorias próprias, como *same-gender-loving* (SGL) ou *down low* (DL) ^26^^,^^37^. Além disso, (v) é possível que, com o passar dos anos, a categoria HSH tenha incorporado certa demarcação identitária *top-down*, ao menos entre os pesquisadores que a utilizam, tornando-a, pois, ou incapaz de funcionar como um mero descritor de comportamento ou impositiva em relação a outras identidades surgidas nos próprios coletivos vulnerabilizados ^38^. Por fim, (vi) o termo HSH, relacionado às campanhas de prevenção de ISTs e de incentivo ao uso de camisinha, reforçou o imaginário de que sexo entre homens está inerentemente vinculado à penetração anal, quando, a depender de especificidades locais, outras práticas são mais recorrentes, como sexo oral e masturbação a dois (*partnered masturbation*) ^38^^,^^39^.

A superação da categoria HSH é, por vezes, associada à proposta de terminologias mais inclusivas. Uma dessas sugestões seria “homens de minorias sexuais” (em inglês, *sexual minority men* ou SMM), já utilizada em algumas pesquisas científicas ^27^. Todavia, ao menos três incômodos tornam a proposta de utilização do termo SMM improvável: (i) o fato de corresponder, novamente, a uma terminologia sem suporte empírico, desvinculada da forma como indivíduos e movimentos sociais respectivos envolvidos referenciam a si mesmos ^40^; (ii) o reforço da categoria “homem” como pressuposta; e (iii) a incorporação da expressão “minoria sexual”, já criticada no meio científico por ignorar sua origem histórica vinculada à condescendência de uma maioria, por reforçar a existência de um padrão majoritário sexual ou por recriar exclusões ao indicar como numericamente inusual comportamentos que, em certas realidades, até então poderiam ser considerados cotidianos ^41^. Outra alternativa a HSH seria “populações sexo e gênero diversas” (em inglês, *sexual and gender diverse population* ou SGD), que considera a multiplicidade de pessoas com identidades, experiências de vida e de opressão interseccionadas ^42^^,^^43^^,^^44^. Embora também incorra em algumas das críticas dirigidas a SMM, a expressão SGD parece avançar na proposta de considerar as orientações sexuais e as identidades de gênero como categorias relacionadas e, portanto, sujeitas a uma abordagem integrada, o que pode ser ainda mais adequado nos estudos destinados a contribuir para macropolíticas de saúde.

Como conclusão parcial, pois, as recomendações de saúde pública relativas a ISTs devem não só superar a utilização da expressão HSH, mas também assumir o compromisso de considerar a identidade de gênero e a orientação sexual das pessoas como categorias relacionais necessárias para se garantir a atenção à saúde. Assim, embora as comunicações destinadas a público amplo possam se valer da expressão SGD, pesquisas científicas e formulários de atendimento precisam registrar tanto a identidade de gênero quanto a orientação sexual por autodeclaração dos pacientes, a fim de que um leque de políticas específicas interseccionais possa ser desenvolvido, com ênfase nas vulnerabilidades mais estigmatizadas.

## A solução microagressiva

Uma última microagressão na breve fala do Diretor-geral da OMS precisa ainda ser mencionada: a recomendação de que HSH reduzissem o número de parceiros sexuais e reconsiderassem o sexo com novos parceiros. Antes de tudo, é preciso esclarecer que nos debates relativos à prevenção de ISTs e, em especial, HIV/aids entre adolescentes e jovens, as estratégias de se evitar a prática de sexo, adiar a iniciação sexual ou reduzir o número de parceiros sexuais são consideradas gradações diversas do conceito amplo de abstinência ^45^, termo que será utilizado neste subitem. Com relação à varíola dos macacos, há dois tipos de abstinência envolvidos: o primeiro é recomendado, de modo geral, para as pessoas contaminadas durante a fase ativa da infecção e logo após a recuperação. Tal recomendação se justifica tanto pela identificação da presença do MPXV no fluido seminal ^46^, ainda que não se possa atestar com precisão o contágio por essa via, quanto pela comprovação de que a relação sexual favorece a transmissão viral, o que se dá pelo contato direto com as erupções e lesões cutâneas, pelo contato face a face com as secreções respiratórias e pelo toque em objetos previamente infectados ^7^. Por esse motivo, a abstinência na fase ativa da infecção por parte dos contaminados foi recomendada pela OMS ^4^ e pelo Centro Europeu de Prevenção e Controle de Doenças (ECDC) ^47^. No mesmo sentido, e por precaução adicional, os órgãos de saúde pública do Reino Unido aconselharam que a abstinência fosse ainda mantida por oito semanas após a recuperação ^48^. O outro tipo de abstinência relacionada à varíola dos macacos foi mencionado por Tedros Ghebreyesus na coletiva de imprensa: sugerido para um grupo específico de pessoas apenas em razão de seu comportamento sexual específico, o fato de serem HSH, mesmo que não diagnosticadas pela infecção.

A diferença entre os dois tipos de abstinência, bem como sua pertinência, já havia causado ruído de comunicação nas políticas preventivas de mpox entre especialistas do Departamento de Saúde de Nova Iorque (Estados Unidos) em junho de 2022. Don Weiss, epidemiologista e Diretor do Escritório de Doenças Transmissíveis, após criticar a chefia do departamento por não recomendar a abstinência para HSH, acabou vindo a público esclarecer, inclusive em audiência pública do Comitê de Saúde vinculado ao Conselho Municipal de Nova Iorque ^49^, que criticara especificamente o fato de as recomendações não indicarem a importância da abstinência sexual para pessoas contaminadas. Mesmo assim, o porta-voz do Departamento de Saúde, Patrick Gallahue, emitiu uma nota pública na qual destacava que, “*por décadas, a comunidade LGBTQ+ teve sua vida sexual dissecada, prescrita e proscrita de inúmeros modos, especialmente por pessoas cis e heterossexuais*” ^49^, razão por que as orientações dadas no passado estavam sendo revistas, dentre as quais, a recomendação estritamente baseada na abstinência sexual, que “*historicamente se mostrou malsucedida na prevenção da transmissão de ISTs*”, tornando-se “*um legado vergonhoso*” ^50^. Dois pontos nessa nota merecem destaque: o receio de condutas que possam fomentar a estigmatização de pessoas sexo e gênero diversas e a ineficácia da abstinência como método de prevenção de ISTs.

Com relação à estigmatização, há sempre o risco de que infecções transmissíveis venham acompanhadas de discursos baseados na patologização do outro, supostamente considerado anormal, e no reforço do parâmetro normativo da pretensa normalidade do emissor do discurso. No caso da varíola dos macacos, ela reativou três tipos de estigmas que, em geral, aglutinam os processos de *labeling*^51^^,^^52^^,^^53^: (i) o estigma tribal ou de origem, que atrela determinada característica a um grupo social geograficamente determinado e, por consequência, a todos os seus integrantes - basta lembrar que, até 2022, as variantes I e II do MPXV eram denominadas, respectivamente, de variante da Bacia do Congo ou da África Central e de variante da África Ocidental, bem como que grande parte das imagens veiculadas nas reportagens dos meios de comunicação europeus sobre as lesões causadas pela varíola dos macacos eram de pacientes negros de origem africana ^54^^,^^55^ -; (ii) o estigma corporal abominável ou do mal-estar perceptível, que, fruto do regime escópico ocidental ^56^, segrega corpos com deficiência ou sintomas visíveis de enfermidade, exemplificado pelo papel que o sarcoma de Kaposi cumpriu no imaginário do HIV/aids; e (iii) o estigma imoral ou de falha de caráter que remete a tabus comportamentais da sociedade, os quais, em relação a populações LGBTQIA+, costumam ser recorrentes. A recomendação pública e ampliada de redução de número de parceiros por parte de HSH reforça, por exemplo, em via oposta, o discurso hegemônico de pessoas heteronormativas que, vivendo relações monogâmicas, consideram outras formas de exercício do direito à sexualidade e à saúde sexual como desvios de conduta. Não sem motivo, a prescrição da abstinência como método prioritário na prevenção de ISTs é apoiada por grupos conservadores e religiosos.

A chamada abordagem ABC - que, em inglês, refere-se ao tripé abstinência, fidelidade e camisinha (*abstinence, be faithful, correct and consistent condom use*) - permitiu que grupos conservadores e religiosos dos Estados Unidos tratassem do tema das ISTs, sobretudo entre jovens e adolescentes, ao mesmo tempo que difundiam seus valores morais. Programas que defendiam a abstinência como único método válido até o casamento surgiram no governo de Ronald Reagan, alcançaram seu ápice no governo de George W. Bush e só foram extintos no governo de Barack Obama. Aliás, o Plano de Emergência do Presidente dos Estados Unidos para o Alívio da Aids (*The United States President’s Emergency Plan for AIDS Relief* ou PEPFAR), quando criado em 2003 por George W. Bush, vinculava o financiamento de projetos à adoção da abordagem ABC. Essa abordagem, por vezes, era estrategicamente parcial, já que incluía mensagens sobre abstinência e fidelidade para adolescentes menores de 14 anos, sem que, no entanto, fossem distribuídos preservativos ^57^. Em contrapartida, pesquisadores integrados a uma comissão científica sobre bem-estar e saúde na adolescência concluíram, em 2016, que há alta evidência de que uma educação exclusivamente baseada na abstinência é inefetiva na prevenção de ISTs, razão por que não é recomendável ^58^. Alguns estudiosos chegaram a questionar a validade do estudo, afirmando ser evidente a eficácia da abstinência e da fidelidade na transmissão de ISTs, por mais impactante que seja a recomendação. Usando como exemplo o combate ao tabagismo, cuja medida mais eficaz para se evitar comorbidades é a suspensão total e imediata do consumo de cigarros e afins por quem seja consumidor, eles defenderam que o mesmo método deve ser utilizado para questões relativas à obesidade e à sexualidade ^45^. Porém, não é possível confundir eficácia, efeitos de uma intervenção sob condições ideais e controladas, com efetividade, efeitos dessa mesma intervenção em situação do mundo real. Combater a transmissão de ISTs com abstinência pode ser eficaz, mas jamais eficiente ^59^. Afinal, considerar que a vida sexual possa ser suspensa ou suprimida, bem como possa ser satisfeita por um número reduzido de parceiros, é recomendação que parte de uma vivência específica da sexualidade com pretensões de universalização. Tal recomendação, assim, pode acabar negando legitimidade às expressões de gênero e aos projetos de vida distintos do padrão majoritário e reforçando a estigmatização e a responsabilização da pessoa infectada, comprometendo a lógica da solidariedade social que inspira os programas de atenção integral à saúde.

Como conclusão parcial, as recomendações de saúde pública relativas a ISTs devem considerar o direito de cada pessoa expressar sua sexualidade e gozar de saúde sexual dentro de uma estrutura de proteção contra a discriminação ^60^, o que significa: (i) considerar a diversidade dos modos de vivência da sexualidade, sem que seja reforçado um paradigma hegemônico; (ii) tratar a sexualidade e a vivência sexual em linguagem positiva, considerando-as não só como manifestação cotidiana dos sujeitos e seus corpos, mas também como um direito; (iii) diferenciar a abstinência entre pessoas na fase ativa da infecção ou no período imediato à recuperação e pessoas que apenas se enquadram entre os destinatários prioritários dos alertas de saúde; (iv) evitar a sugestão irrestrita de redução do número de parceiros, especialmente porque a suposição de um número adequado de relações só se justifica quando baseada no comportamento heteronormativo majoritário; (v) suprimir mensagens que reforcem a concepção social de que ISTs são uma punição justa por um suposto desregramento sexual, o que acontece quando se descreve comportamentos ou práticas sexuais não normativas de modo detalhado para o público amplo.

## Considerações finais

A experiência com as recomendações de saúde pública sobre mpox da OMS e, em especial, as declarações públicas de seu Diretor-geral motivaram o presente estudo a buscar alguns parâmetros de comunicação que, simultaneamente, garantam as políticas de prevenção de ISTs sem estigmatizar grupos vulnerabilizados. As sugestões, sumarizadas ao fim de cada um dos itens acima, podem ser consolidadas da seguinte forma: (i) diferenciar as divulgações endereçadas ao público geral, focadas nas características epidemiológicas da infecção, das destinadas às populações predominantemente contaminadas e sujeitas a um maior grau de vulnerabilidade social, para as quais devem ser dadas informações específicas, observando a diversidade de perfil dos infectados, focando nos locais usualmente frequentados para a prática de sexo casual, nos aplicativos de relacionamento e nos serviços ambulatoriais de atendimento a ISTs, aliando-se às associações e organizações não governamentais que mantenham com tais populações um contato mais amigável e menos disciplinar que os órgãos estatais; (ii) superar a utilização da expressão HSH para, nas comunicações destinadas à ampla audiência, utilizar a expressão SGD, mantendo-se o procedimento de registrar, nas pesquisas científicas e nos formulários de atendimento, tanto a identidade de gênero quanto a orientação sexual por autodeclaração dos pacientes; (iii) evitar mensagens que abordem a sexualidade de modo negativo, reforcem uma vivência sexual majoritária ou, ainda, gerem uma responsabilização socialmente punitiva do infectado, excluindo, pois, das recomendações irrestritas, voltada para o público amplo, a sugestão de abstinência sexual parcial, relativa à redução do número de parceiros, ou de abstinência sexual total, exceto para os casos de pessoas na fase ativa da infecção ou no período imediato à recuperação.

O aperfeiçoamento das recomendações públicas de saúde, desse modo, não só respeitaria “*o direito ao padrão mais alto alcançável de saúde física e mental, sem discriminação por motivo de orientação sexual ou identidade de gênero*”, previsto no Princípio 17 de Yogyakarta e elaborado por especialistas em direitos humanos de diversos países reunidos na Indonésia em 2006 ^61^, e o princípio da dignidade da pessoa humana, previsto na *Constituição Federal* brasileira de 1988 ^62^, como avançaria na compreensão de que o direito à sexualidade e à saúde sexual deve englobar igualmente os meios, inclusive comunicacionais, de garanti-lo.
